# Graph representation of high-dimensional alpha-helical membrane protein data

**DOI:** 10.1186/1756-0381-6-21

**Published:** 2013-12-02

**Authors:** Steffen Grunert, Dirk Labudde

**Affiliations:** 1 Department of Mathematics, Sciences, Computer Science, University of Applied Sciences Mittweida, Technikumplatz 17, Mittweida 09648, Germany

**Keywords:** Membrane proteins, Motifs, Graph, Architecture

## Abstract

**Background:**

In genomics and proteomics, membrane protein analysis have shown that such analyses are very important to support the understanding of complex biological processes. In Genome-wide investigations of membrane proteins a large number of short, distinct sequence motifs has been revealed. Such motifs found so far support the understanding of the folded membrane protein in the membrane environment. They provide important information about functional or stabilizing properties. Recently several integrative approaches have been proposed to extract meaningful information out of the membrane environment. However, many information based approaches deliver results having deficits of visualisation outputs. Outgoing from high-throughput protein data analysis, these outputs play an important role in the evaluation of high-dimensional protein data, to establish a biological relationship and ultimately to provide useful information for research.

**Results:**

We have evaluated different resulting graphs generated from statistical analysis of consecutive motifs in helical structures of the membrane environment. Our results show that representative motifs with high occurrence in all investigated protein families are responsible for the general importance in alpha-helical membrane structure formation. Further, motifs which often occur with others in their function as so called “hubs” lead to the assumption, that these motifs constitute as important components in helical structures within the membrane. Otherwise, consecutive motifs and hubs which show a high occurrence in certain families only can be classified as important for family-specific functional characteristics. Summarized, we are able to bridge our graphical results from high-throughput analysis of membrane proteins over networking with databases to a biological context.

**Conclusions:**

Our results and the corresponding graphical visualisation support the understanding and interpretation of structure forming and functional motifs of membrane proteins. Our results are useful to interpret and refine results of common developed approaches. At last we show a simple way to visualise high-dimensional protein data in context to biological relevant information.

## Introduction

Proteins are the main catalysts, structural elements, signalling messengers, molecular machines of biological tissues and essential for many fundamental biological processes within organisms [1]. Fundamental biological processes depend on membrane proteins. Membrane proteins fall into a class of proteins whose molecules are attached to or are associated with the membrane of a cell. A variety of biological functions are accomplished by these membrane proteins, such as signal and energy transduction, nutrient transport, the maintenance of ion concentration, ligand binding, and cell adhesion [2], thereby facilitating their functional importance in many biological processes [3]. Many fundamental cellular processes involve protein–protein interactions, and membrane proteins are no exception. Comprehensively identifying complexes is important to systematically defining protein function [1], and hints about the function of an unknown protein can be obtained by investigating its interaction with other proteins of known function. Nervous excitement, oxygen supply, energy balance, immune response and the transmission of signals within cells and from cell to cell are the essential of membrane proteins. E.g. membrane proteins form specific receptors on the cell surface and serve as the communication interface between the cell’s external and internal environment [4]. Hormones and other neurotransmitters can bind to these and thereby causing the cell to certain reactions. They play a fundamental role within cellular and physiological processes. Membrane proteins perform different tasks. They can be involved as transport proteins, compound molecules, receptors or enzymes. As structure proteins they determine the cell’s design and ultimately the quality of tissues and the whole body. The ion concentration regulation in the cell and the excitability of nerves and muscles are functions of a membrane protein as ion channel. As transport proteins, they handle vitally important substances like e.g. glucose which is essential for the energy supply in the whole body. The identification of such protein complexes and interactions is valuable, since, on the one hand, detailed information of the function of an unknown membrane protein can be obtained by analysing its interactions with proteins of known function. On the other hand, biological processes can be comprehended as a dynamically fluctuating system, whereby the biological role of the unknown membrane protein can be defined more precisely [1,5]. In summary, membrane proteins convey the material and information transfer between cells and organ systems. Functional intact membrane proteins are indispensable for human health. They are aim of a large number of drugs and pharmacologically active substances. However, if they exhibit specific defects, they lead to the formation of many known diseases like e.g. Alzheimer’s, Parkinson’s, diabetes insipidus, hereditary deafness, cystic fibrosis, retinitis pigmentosa or cancer [6-8].

**Figure 1:**
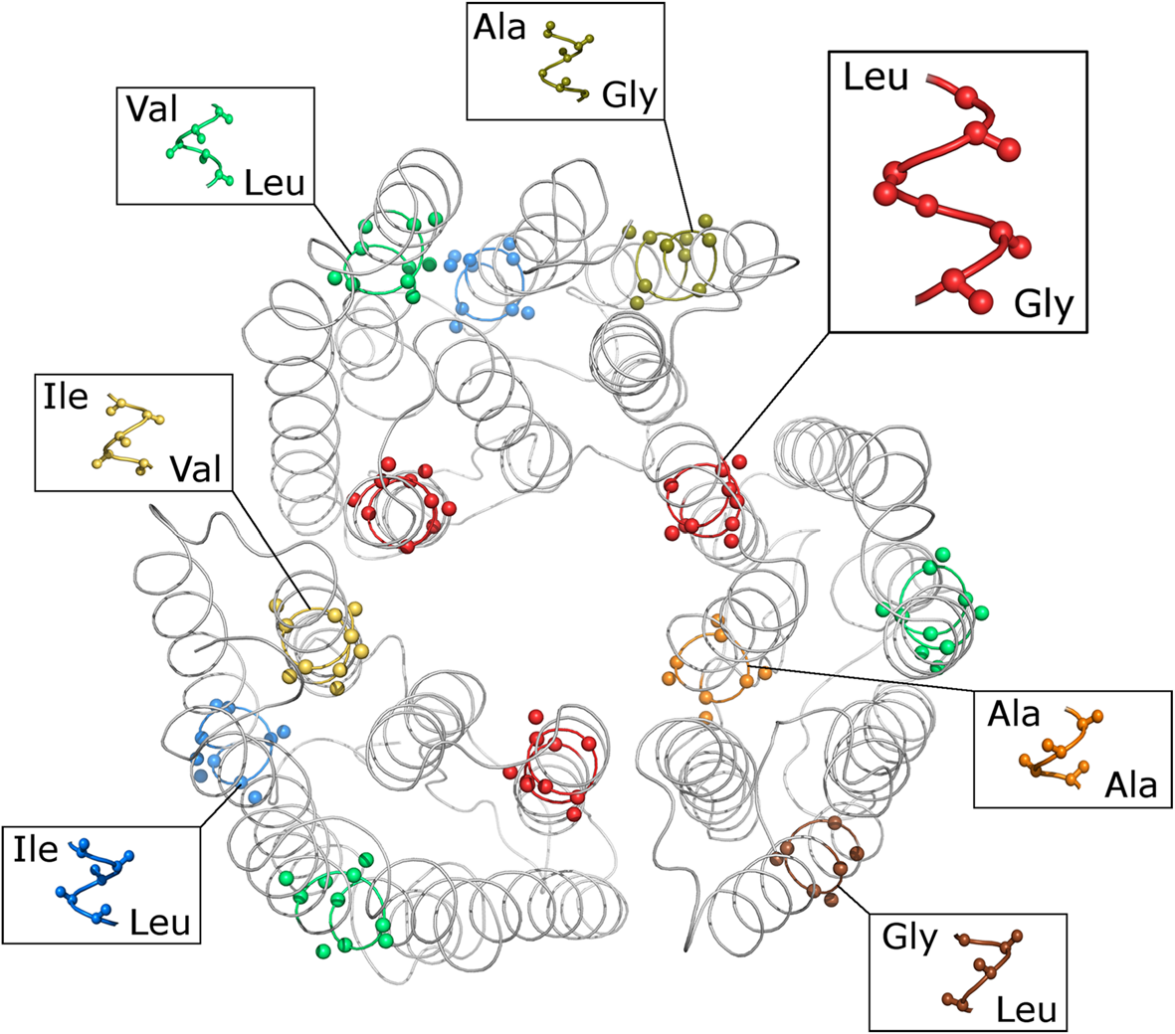
**Motif Examples.** In the bacteriorhodopsin trimer (PDB-Id: 1brr), seven motif examples are present. Each motif can be written in a regular-expression like XYn, where X and Y are amino acids separated by n -1 highly variable positions. For example the LG5 motif occurrence (highlighted in red) corresponds to a pair of leucine (Leu) and glycine (Gly) residues which are separated by four amino acids.

In conjunction with genome-wide investigations, previous works have been engaged in analysing of classified poly-topic membrane protein families. For example the research of Y. Liu, D. M. Engelman and M. Gerstein observed the amino acid distribution of TM helices in their work of computational genomic analysis of membrane protein families [9]. The abundance of conserved motifs in the transmembrane helix regions in these families has been carried out. The structural analyses in terms of patterns of protein folding have been useful in revealing functional and evolutionary relationships and supporting the understanding how a protein folds in the membrane environment. Here, Liu and colleagues studied the most widely discussed GxxxG and GxxxxxxG motif, and found that they tend to be associated and relatively conserved within transporter/channel-like membrane proteins [9]. Structural studies confirmed that the GxxxG motif plays an important part in mediating helix-helix interactions [9-13]. Eventually, information about discriminative motifs can be statistically interpreted in a membrane protein sequence [9,11]. Besides, a logOdd-profile generation approach by Grunert and colleagues [14] addresses the separation task of discriminative sequence motifs by determination of the residue conservation at each variable motif position. Based on such logOdd-profiles a currently yet unpublished approach addresses the prediction of helical ranges of membrane proteins by a given protein sequence. This confirms and includes information about that a specific three-dimensional protein structure depends on the information stored in the corresponding amino acid sequence. Thus sequence motif analysis can be helpful in a number of approaches and applications, e.g. the investigation of mutant proteins and potential effects of mutagens. Independent of their functionality and possible structure forming properties, different motif examples are illustrated in Figure 1, which shows seven motifs in the bacteriorhodopsin trimer (PDB-Id: 1brr).

The unsolved problem how a protein folds and sequence homology are related can be better understood by sequence motif analyses. Thus, the enormous increase of membrane protein data and protein structures requires the handling of such high-dimensional biological data. In this work, our novel statistical approach shows which motifs contribute fundamentally to be involved as structural or functional sequence parts. Useful graph visualisations will fill the lack of high-throughput protein data analysis and evaluation. Here, we will reveal functional and structural relationships of sequence motifs. Summarized, we inspect structural and functional aspects of sequence motifs within the field of membrane proteins, largely from a computational point of view.

## Materials and methods

### Used membrane protein family datasets

As first step of our analysis different datasets were obtained. Two of them were derived from the Pfam database [15]. The first dataset (DS1) consists of 32 membrane protein families which include 2511 proteins with domains of unknown functions (DUF) as listed below.

[PF09767, PF09834, PF09842, PF09843, PF09852, PF09858, PF09874, PF09877, PF09878, PF09879, PF09880, PF09881, PF09882, PF09900, PF09913, PF09925, PF09945, PF09946, PF09971, PF09972, PF09973, PF09980, PF09990, PF09991, PF09997, PF10002, PF10011, PF10067, PF10080, PF10081, PF10097, PF10101]

The second dataset (DS2) consists of 11 membrane protein families with 15644 proteins and 160 known structures as listed below.

[PF00001, PF00002, PF00003, PF00664, PF00939, PF01490, PF02932, PF05602, PF06472, PF06814, PF10192]

After the datasets have been obtained, non-redundant sequences from DS1 and DS2 were generated. To avoid generating misguiding statistics by including identical or highly similar sequences, CD-HIT [16] and BlastClust [17] were applied using by a threshold setting of 25% and 60% respectively. Further, we determined the helical structures in transmembrane regions of the proteins to be investigated, using the TMHMM Server v. 2.0 [18]. Basically, TMHMM performs a prediction of intra/extra-cellular regions and integral membrane helices starting from sequence. Additionally, the probability of the prediction is given for each residue as well. According to the obtained results from TMHMM, a topological state was assigned to each residue. A residue was assigned as ‘TM’ if the posterior prediction probability of this residue being a part of a membrane helix and has been found to be greater than 90%. If the posterior prediction probability of the residue has been found to be greater 90% for extra/intra-cellular prediction, the residue was assigned as ‘nTM’.

### Sequence motif extraction

Generally, proteins are large biological molecules they fold into a three-dimensional structure, which is determined by the protein sequence (primary structure) which consists of one or more chains of the 20 canonical amino acids. In the current work only ‘TM’ sequence information was used for our analysis. In this context, short sequence motifs have been extracted which contribute to build the membrane protein structure in the ‘TM’ environment. Each extracted motif can be written in a generalized, regular expression-like form of XYn, where X and Y correspond to amino acids separated by n-1 highly variable positions.

**Figure 2:**
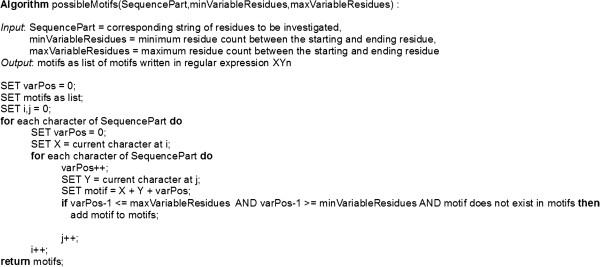
**Motif Extraction.** The present pseudo code describes a naive algorithmic procedure of variable word extraction from any string. We have applied this procedure to the context of motif extraction out of different protein sequence information. Ultimately, this algorithm returns the starting X amino acid at each protein sequence position i and the ending amino acid Y at i + n of the corresponding extracted word as representative motif. A resulting list consists of motifs which are all written in the regular expression XYn by n = {4 - 7}. A list without duplicate entries will be updated if the supplied current motif does not exist.

A naive text search algorithm was applied for motif extraction (see Figure 2). Here the algorithm is involved in a step by step window moving process. Beginning from starting position, different defined window sizes lead to several sequence cutouts of matching sizes. Each cutout has been transcribed into the regular expression XYn. More specifically this algorithm returns at each ‘TM’ sequence position i the starting X amino acid and at i + n the ending amino acid Y of the corresponding extracted motif XYn. A resulting list consists of motifs (without duplications) in regular expression XYn form by n={4-7}.

### Topology separation and prediction of discriminative motifs

For later evaluation of our frequently occurring motif combinations, we have predicted the topology state of all motifs extracted from ‘TM’ sequence information. About this prediction task, we will figure out which motif is atypical for the ‘TM’ environment. By using a new straight-forward approach of information extracting and clustering this approach addresses the prediction task by determination of the residue conservation at each variable motif position. At first, all single motif occurrences were identified in the non-redundant DS1 and DS2. Including TMHMM predictions, each motif occurrence was assigned to a topology state as previous elucidated. Subsequently, all variable positions within each motif occurrence were examined more closely. Ultimately for each variable position the relative occurrence of each amino acid at the specified position of each motif was calculated and set into relationship to nature occurrence. Like described in [14], the significance of each resulting probability was applied in a log-odd formula. Log-odd values of variable positions were transformed into a vector which ultimately leads to generated logOdd-profiles (LOPs). Based on this LOPs we are fundamentally able to separate each variable motif position to a topology state and finally to predict the topology state of each motif. This approach is discussed in detail in [14].

### Information extraction and visualisation from motif architectures

**Figure 3:**
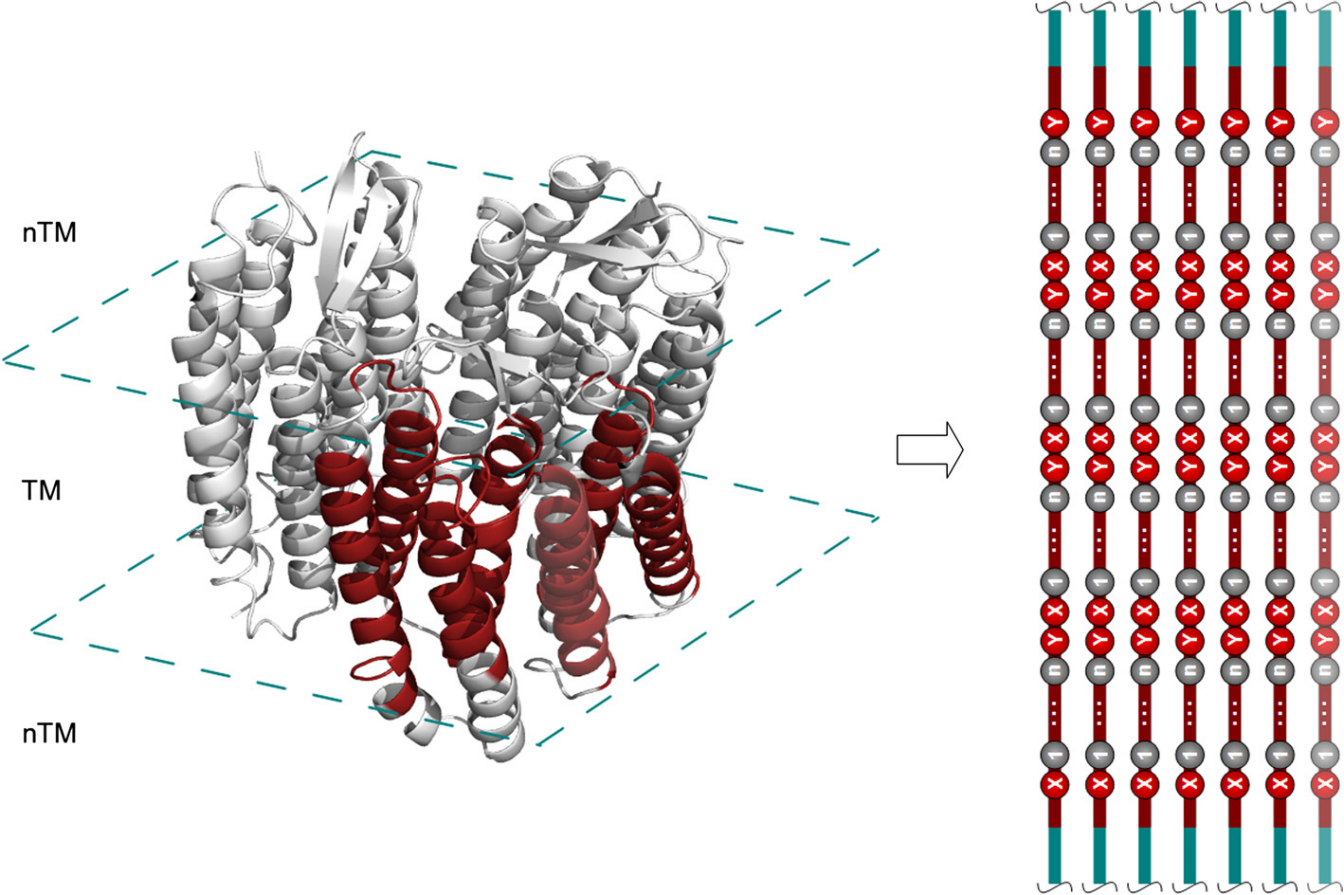
**MA Extraction.** Using of ‘TM’ helical information (red coloured) to create own statistical restrictive frames called motif-architecture (MA). Each MA consists of four consecutive motifs where X and Y of a motif XYn corresponds to one of the 20 canonical amino acids and n-1 defines the length of highly variable positions between X and Y by n = {4 - 7}. In the case of the bacteriorhodopsin trimer (PDB-Id: 1brr), seven transmembrane alpha-helices have been predicted by TMHMM. ‘TM’ helical information was used to search for MAs with strictly defined architecture size of four motifs.

**Figure 4:**
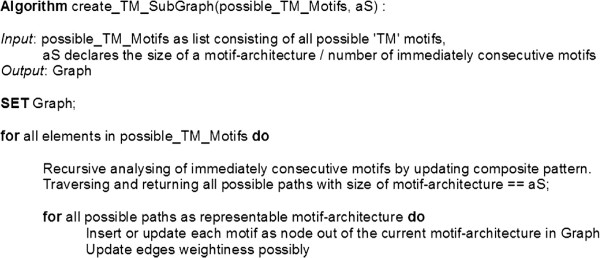
**Graph Creation.** The present pseudo code describes different steps to transform motif-architecture information into a graph structure in a suggestively way. The first step deals with the creation of a parent-child relationship mapped by the common composite pattern. Here, each child is the immediately consecutive motif of the previous within a sequence. The next step handles the final resulting composite which has been traversed and returns all possible paths with our strictly defined size of four consecutive motifs. A motif-architecture was born and transferred into a graph. A representative motif as node will be inserted if the node does not exist or updated if it exists in the graph. The corresponding edge of two nodes will be updated by increasing by one if this connection already exists. This leads to one graph for each ‘TM’ region. All ‘TM’ sub-graphs will be merged into one main graph.

**Figure 5:**
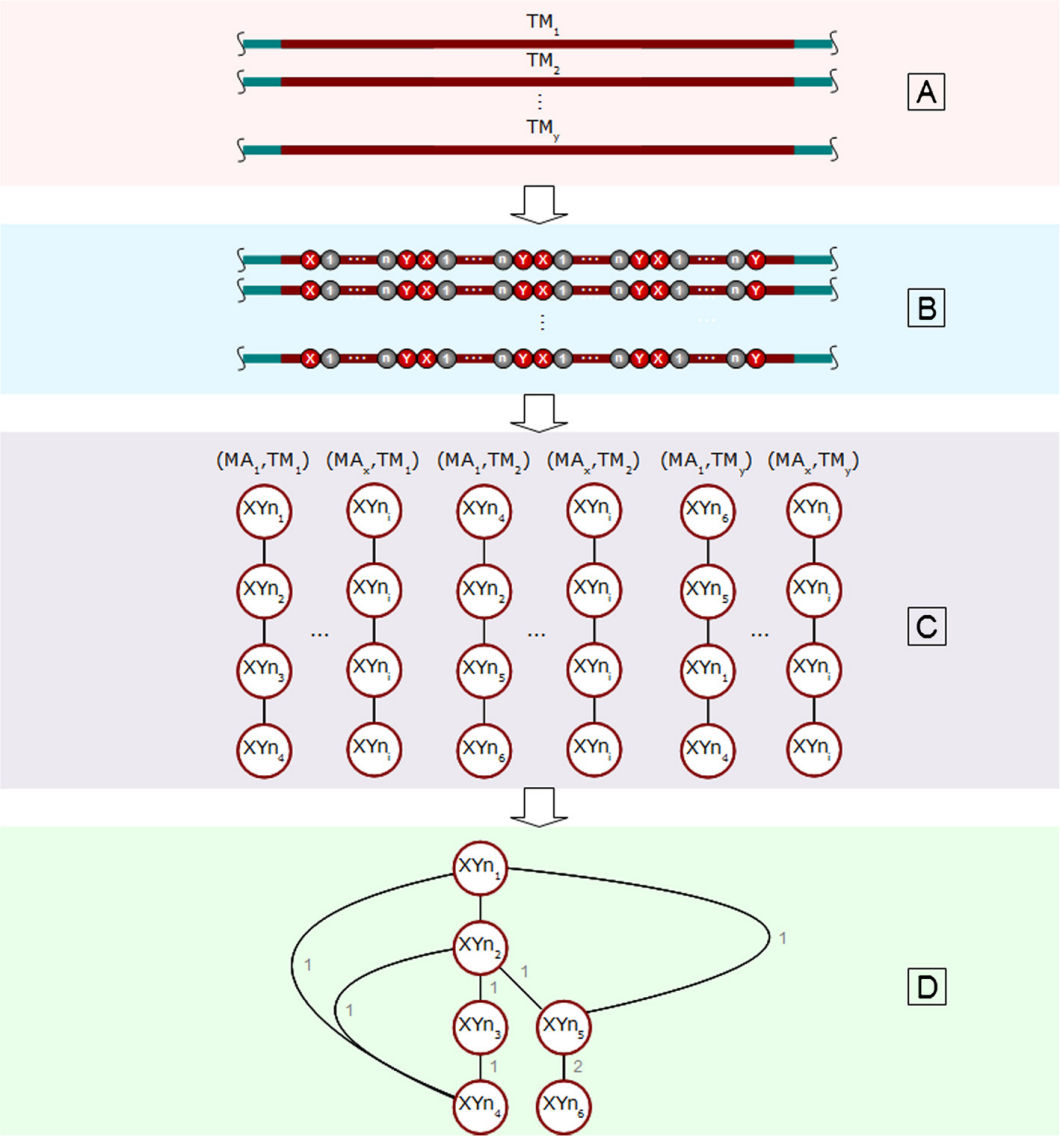
**The Workflow.** The workflow for membrane environment information extraction and transformation. **A:** For each membrane protein, all possible membrane helices have been predicted using TMHMM. Predicted ‘TM’ sequence information is coloured in red and ‘nTM’ in blue. **B:** After deriving ‘TM’ sequence information all possible motifs with n-1 highly variable positions by n = {4 - 7} were determined by using a common naive text search algorithm (Figure 2). Further, for each ‘TM’ sequence part, all possible MAs consisting of four directly consecutive motifs have been detected. **C:** The later applying of useful and powerful algorithms which are involved in the statistical information aggregation assumes, that each detected (MA_*x*_, TM_*y*_) is considered to be a graph structure. This leads to the transfer of each (MA_*x*_, TM_*y*_) into a graph where each motif can be considered as a node connected by a edge to the following node. **D:** Finally, all ‘TM’ sequence part corresponding graphs were merged into one. The edge-weightiness of the already existing source and target nodes were updated by increasing by one. Ultimately, a weighted graph exists for each ‘TM’ sequence part which leads to the final merge process and the resulting graph.

Furthermore, for our statistical analysis of highly occurring consecutive motifs in ‘TM’ regions, a statistical restrictive frame called “motif-architecture” (MA) was defined. In this work a MA specifies that only four directly consecutive motifs are to be considered in each statistical frame. The number of four consecutive motifs depends on the number of ‘TM’ environment occupied residues and the maximum length of a motif defined for this work. In addition directly consecutive motifs means that a motif is ultimately following the previously (Figure 3) without residue gaps between both. Followed by MA analysing from ‘TM’ sequence information a result set with a number of MAs was created. A list of MAs can be assigned to each investigated ‘TM’ region. Relating to further statistical analysis, the decision to apply useful and powerful graph-algorithms causes that each found MA has been considered as a graph structure (see Figure 4). In general, a graph consists of a number of nodes connected by edges. Related to our MA a motif can be considered as a node connected to another node by a weighted edge. The edge weightiness between two nodes depends on the occurrence of edges with same source and target node in all detected MAs. One main graph for each ‘TM’ region has been created by merging all graphs out of the corresponding ‘TM’ list. This leads to the same number of graphs as they are ‘TM’ regions to be analysed. The final step includes the same merging procedure of all ‘TM’-graph to one main-graph included by updating the edge weightiness. So the weightiness of already existing edges was updated by increasing by one. The final main graph includes all motifs as representative nodes connected over weighted edges. By defining an edge weight threshold we are able to reduce the graph by removing less weighted edges and keeping stronger ones. These different steps were applied to DS1, DS2 and selected protein families. This workflow for membrane environment information extraction and transformation is shown in Figure 5.

## Results and discussion

**Figure 6:**
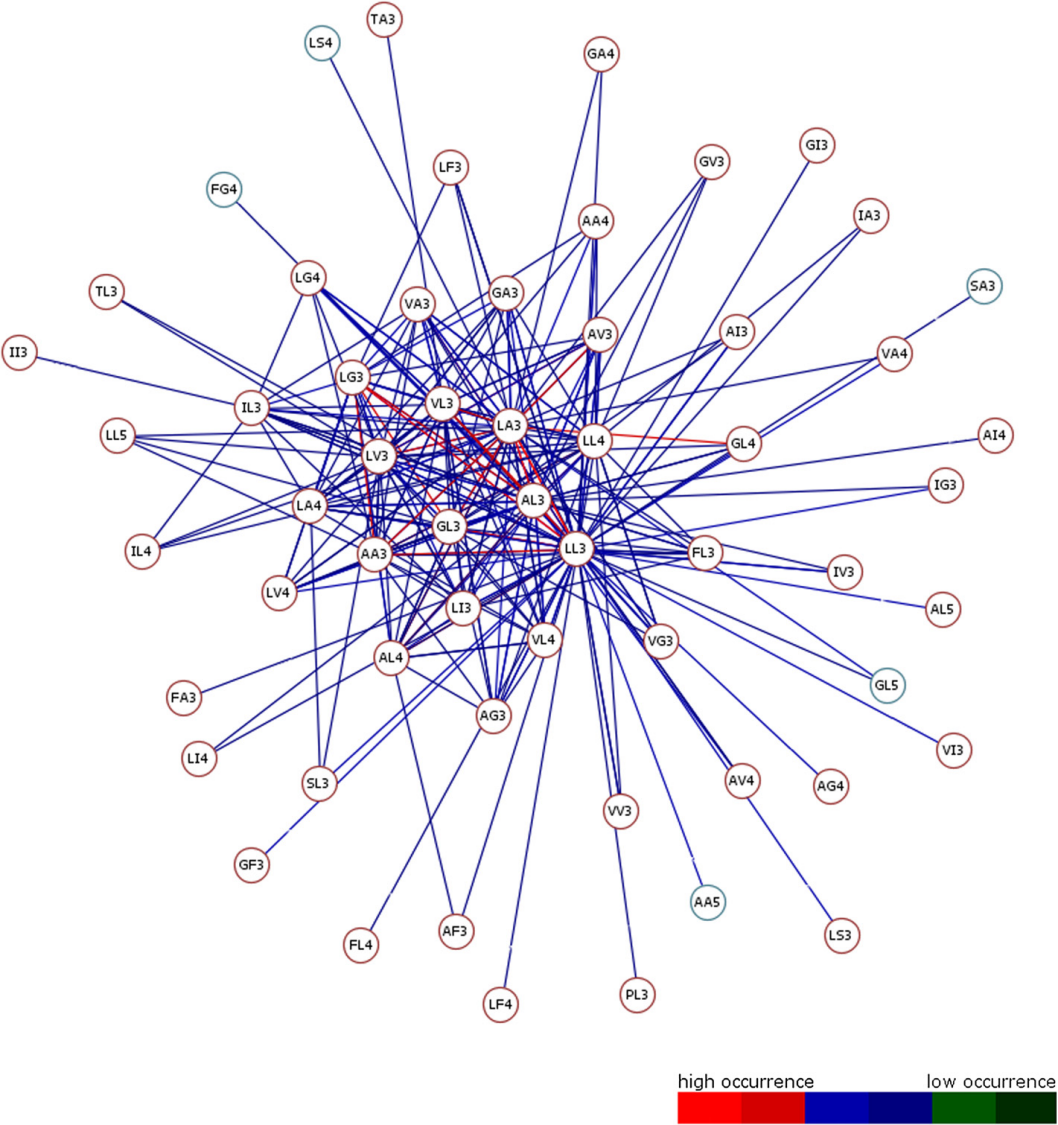
**Result Graph DS1.** The result graph for DS1 generated from TMHMM transmembrane-helical information. After removing less weighted edge connections, the graph is given more clearly. Different nodes are given as representative motifs. Two possible node colours describe the predicted topology state (TM = transmembrane, nTM = none-transmembrane) based on previous work by Grunert and colleagues [14] for each motif closer. This distinguishes TM-typical from TM-non-typical graph present motifs. Coloured weighted edges can be assigned to a occurrence value of the pendant colour-scale. Ultimately, the graph makes clear how often different consecutive motifs occur. Highly occurring motifs are connected with red coloured edges. It is shown that always the same residues are recovered at the starting and ending position of a motif. Here alanine, leucine, glycine or valine are the most involved starting and ending residues which get a great importance in structure forming motifs. Finally, often accrued motifs become apparent in their function as “hub”-motif. For example LL3, LV3, VL3 and AL3 often occur within a MA with other motifs. This leads to the indispensability for building helical regions within the membrane environment.

**Figure 7:**
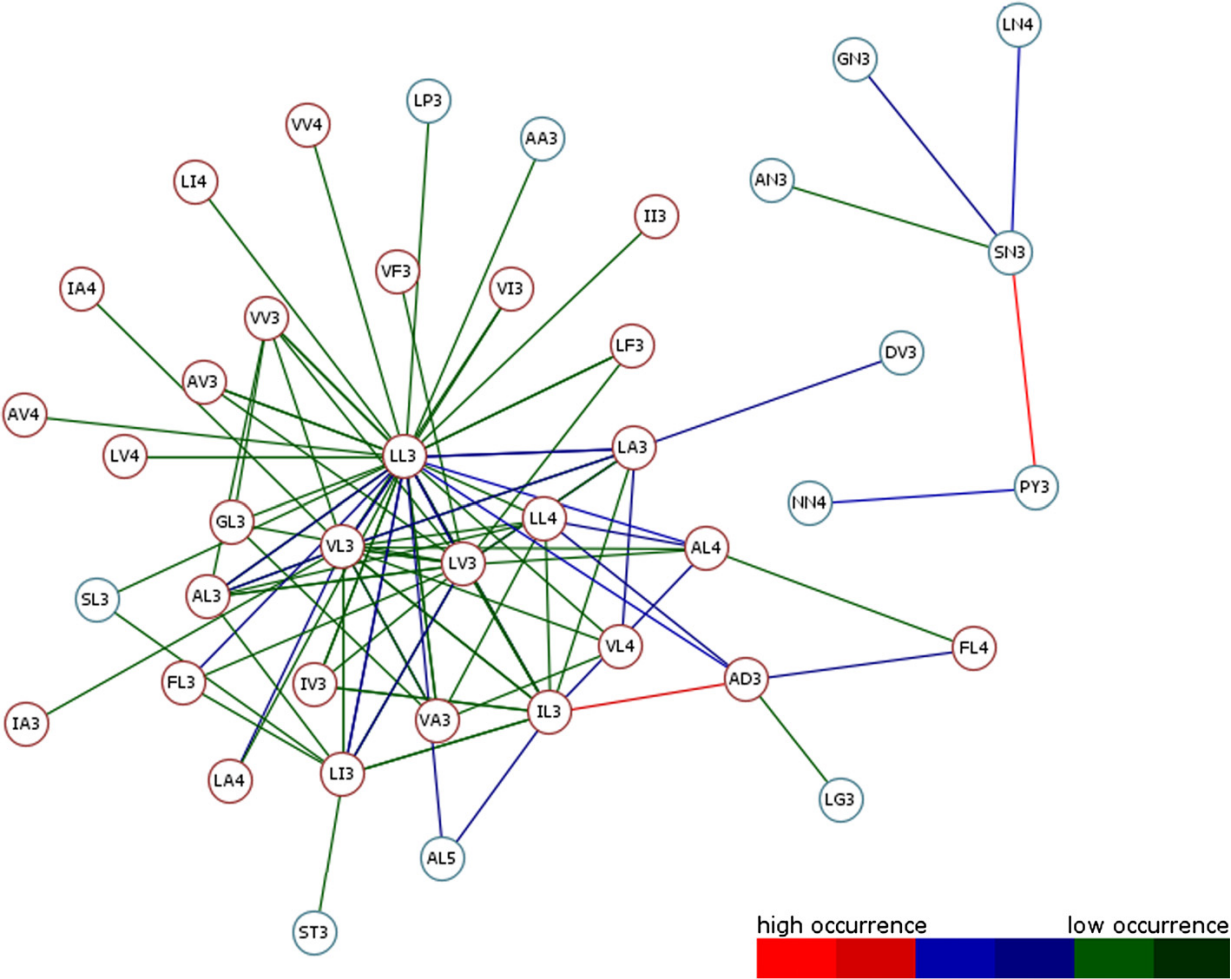
**Result Graph DS2.** The reduced result graph for DS2 generated from TMHMM transmembrane-helical information. After removing less weighted edge connections, the graph is given more clearly. Different nodes are given as representative motifs. Two possible node colours describe the predicted topology state (TM = transmembrane, nTM = none-transmembrane) based on previous work by Grunert and colleagues [14] for each motif closer. This distinguishes TM-typical from TM-non-typical graph present motifs. Coloured weighted edges can be assigned to a occurrence of the pendant colour-scale. Ultimately, the graph makes clear how often different consecutive motifs occur. Highly occurring motifs are connected with red coloured edges. It is shown that always the same residues are recovered at the starting and ending position of a motif. Typical motifs with alanine, leucine, glycine or valine starting and ending residues are the representative structure forming motifs. Finally, often accrued motifs become apparent in their function as “hub”-motif. For example LL3, LV3, VL3 and AL3 often occur within a MA with other motifs. This leads to the indispensability to build helical regions within the membrane environment. The graph also shows motifs atypical for membrane environment. E.g. the topology prediction of SN3 and PY3 to ‘nTM’ state can be traced back to more ‘nTM’ conservation in evolution. This leads to the assumption, that these motifs are functionally important and relevant for family-specific functional characteristics of DS2.

The high throughput analysis of membrane protein families obtained by previous described steps returns different result graphs. Useful information about frequently occurring consecutive motifs has been ascertained for all investigated membrane protein families of DS1 and DS2. The resulting graphs of both datasets are shown in Figures 6 and 7. Edge colourations illustrate heavily or less weighted edge connections which arise from high common occurrences of the edge ending source and target motif. Each edge colour can be assigned to a colour-range of the graph pendant colour-scale. In the course of this each range corresponds to a range of edge weight values. Removing of less weighted edges minimizes the graphs to clearly arranged structures. Different motifs emerge to structure forming components considered to all protein families of an investigated data set. Also apparent is the positioning of graph centred motifs, they often occur together with others (e.g. LL3, LV3, VL3, IL3 and AL3). This leads to the assumption that these so called “hub”-motifs constitute important components within a MA and thus in helical regions. Depending on how a alpha-helical structure is constructed, these motifs are required for filling the gaps in the physical and structural context. This hypothesis confirms previous work of [19] and colleagues who dealt with the projections of three-dimensional structures of alpha-helices into two-dimensional images which they called helical wheels. Their results stating that Ala, Val, Leu or Ilu residues are important members of helical wheels also relate to our representative hub-motifs. So both result graphs (Figure 6 and 7) are showing residues which can always be recovered at X and Y of a motif XYn.

**Figure 8:**
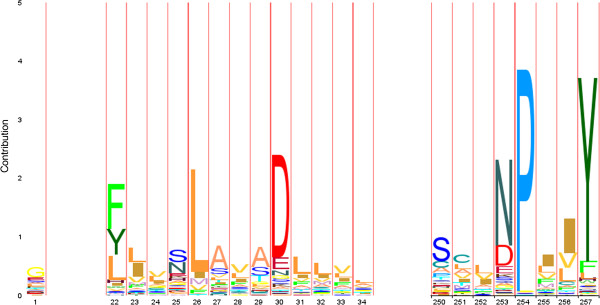
**WebLogo PF00001.** Excerpt from a WebLogo for 7tm_2 (PF00001) transmembrane receptor rhodopsin family, derived from an entropy based Profile Hidden Markov Model (pHMM)-alignment approach by [20] and colleagues. FL4-AD3 and SN3-PY3 are examples of consecutive motifs which are also present in the DS1-graph (Figure 10).

**Figure 9:**
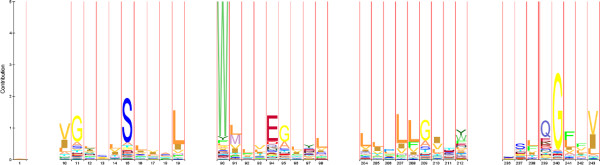
**WebLogo PF00002.** Excerpt from a WebLogo for 7tm_2 (PF00002) transmembrane receptor rhodopsin family, derived from an entropy based Profile Hidden Markov Model (pHMM)-alignment approach by [20] and colleagues. GL3-SL4, WE4-GL3, LL4, LT3 and NQ3-GI3 are examples of consecutive motifs which are also present in the DS2-graph (Figure 11).

**Figure 10:**
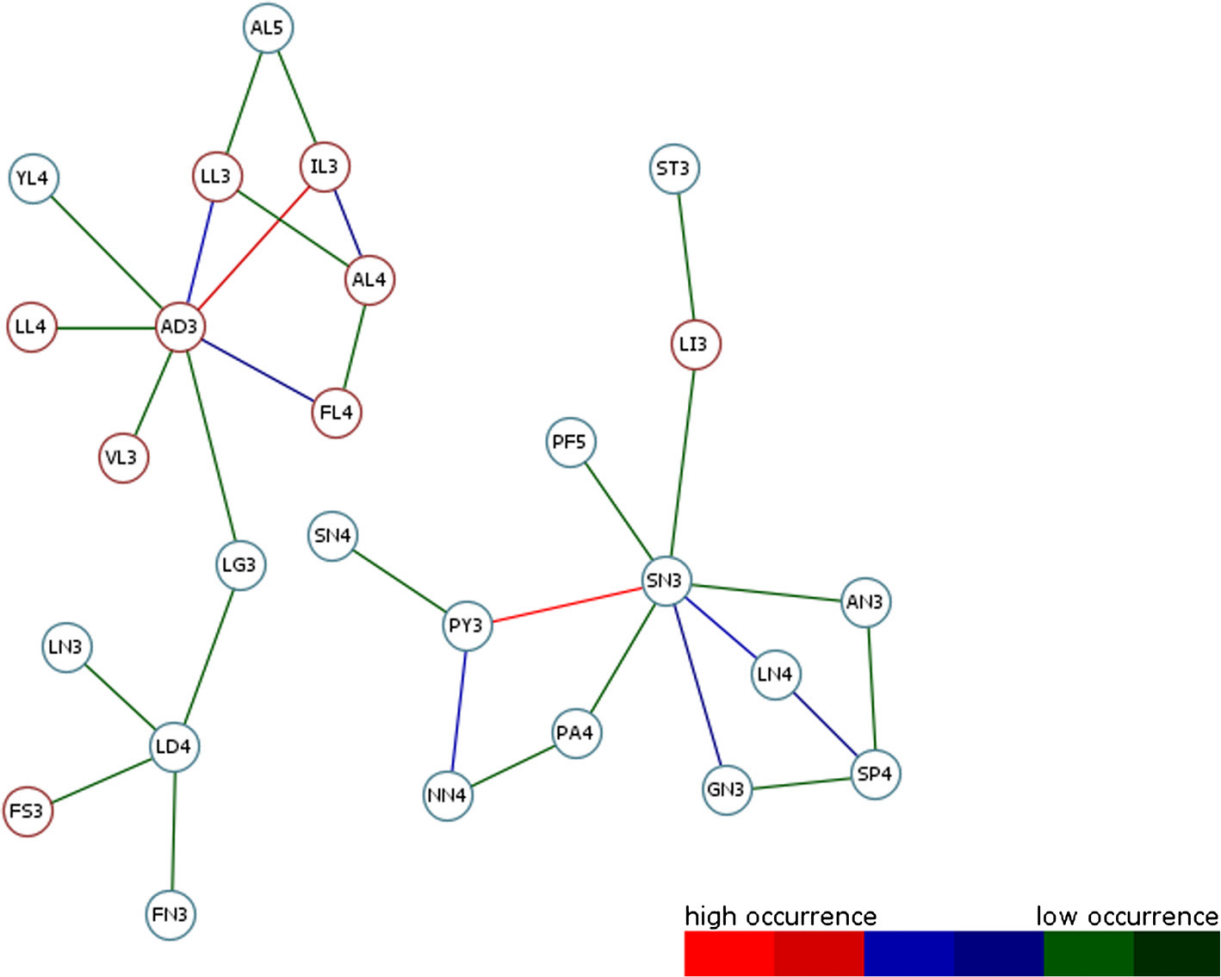
**Result Graph PF00001.** The reduced result graph for 7tm_1 (PF00001) transmembrane receptor rhodopsin family generated from TMHMM transmembrane-helical information. After removing less weighted edge connections, the graph is given more clearly. Different nodes are given as representative motifs. Two possible node colours describe the predicted topology state (TM = transmembrane, nTM = none- transmembrane) based on previous work by Grunert and colleagues [14] for each motif closer. This distinguishes TM-typical from TM-non-typical graph present motifs. Coloured weighted edges can be assigned to a occurrence of the graph pendant colour-scale. Ultimately, the graph makes clear how often different consecutive motifs occur. Highly occurring motifs are connected with red coloured edges. Here, SN3-PY3 are the most common consecutive motifs. This occurrence is specific for this family and can be responsible for possible functional or structural protein features. Networking with existing biological databases like PROSITE [21-24] delivers important information about protein domains, families and functional sites as well as associated patterns and profiles to identify them. In relation to SN3-PY3, these motifs are involved in the consensus pattern of retinal binding sites. Retinal binding site matching PDB structures are e.g. 1BOJ, 1BOK, 1F88, 1GZM.

**Figure 11:**
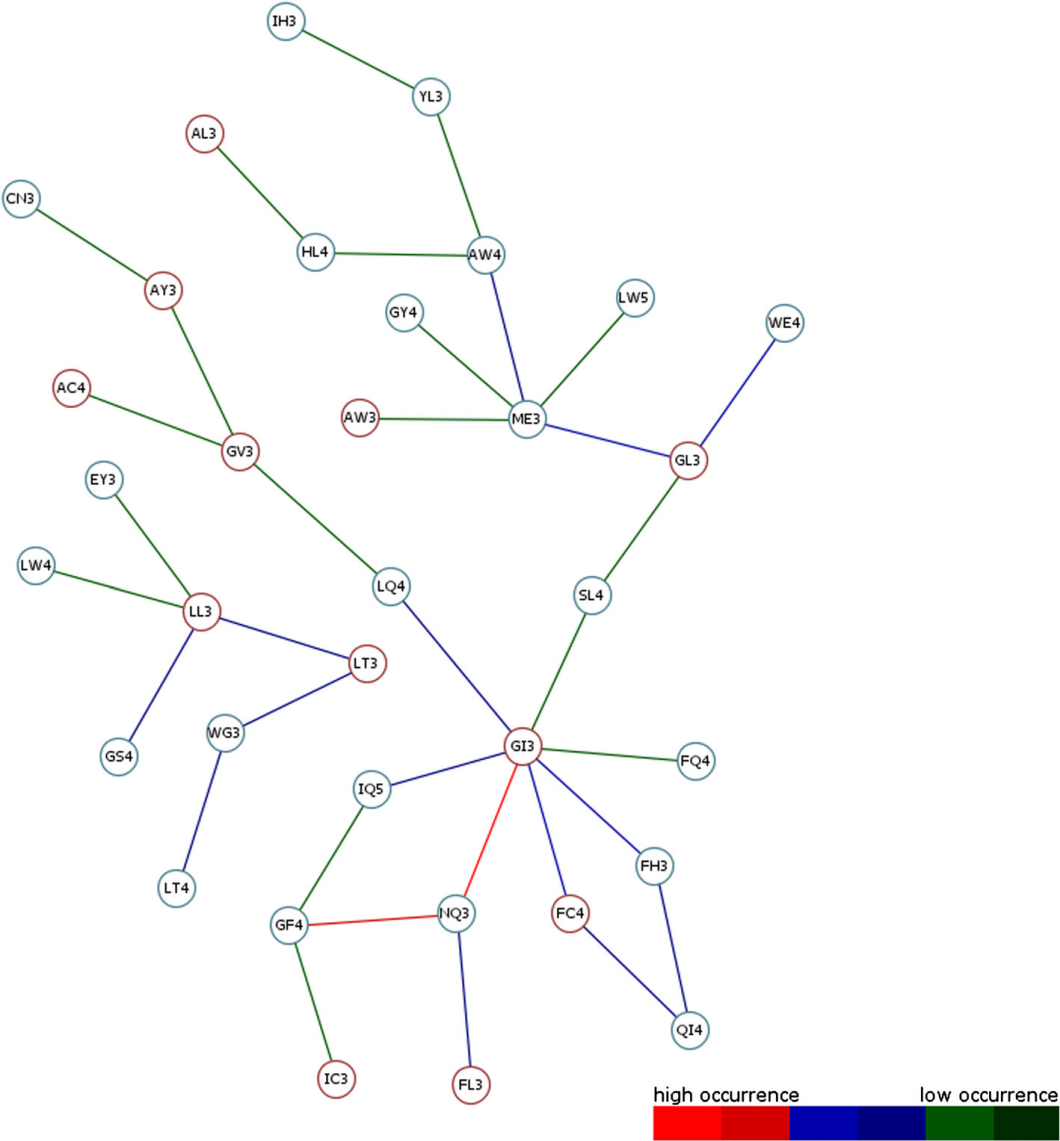
**Result Graph PF00002.** The reduced result graph for 7tm_2 (PF00002) transmembrane receptor rhodopsin family generated from TMHMM transmembrane-helical information. After removing less weighted edge connections, the graph is given more clearly. Different nodes are given as representative motifs. Two possible node colours describe the predicted topology state (TM = transmembrane, nTM = none- transmembrane) based on previous work by Grunert and colleagues [14] for each motif closer. This distinguishes TM-typical from TM-non-typical graph present motifs. Coloured weighted edges can be assigned to a occurrence of the graph pendant colour-scale. Ultimately, the graph makes clear how often different consecutive motifs occur. Highly occurring motifs are connected with red coloured edges. Here, NQ3-GI3 are the most common consecutive motifs. This occurrence is specific for this family and can be responsible for possible functional or structural protein features. Networking with existing biological databases like PROSITE [21-24] delivers important information about protein domains, families and functional sites as well as associated patterns and profiles to identify them. In relation to NQ3-GI3, these motifs are involved in the consensus pattern of retinal binding sites. Retinal binding site matching PDB structures are e.g. 1ET2, 1ET3.

Further, different coloured Nodes are recognizable. Here, red Nodes were predicted to be part of ‘TM’ region and blue ones to be part of ‘nTM’ by determination of the residue conservation at each variable motif position of the given data sets like described in section “Topology separation and prediction of discriminative motifs”. Based on this, ‘TM’ non-typical motifs have been predicted in both result graphs. Such motifs can indicate, that they may be involved in special functions. On closer inspection of the DS2 result graph (Figure 7), a highly weighted edge catches the eye. This edge represented by the SN3 and PY3 motif is to be silhouetted against all other edges. Here the motif building start residue serine (S) consists of aliphatic hydroxy-groups and corresponds formal to a hydroxylated alanine (A). By hydroxylation serine is more hydrophilic than alanine. The motif end residue asparagine (N) as uncharged derivative of aspartate plays an important role in covalent protein modifications because carbohydrate residues may be attached to this amino acid. All these properties are not typical for helical structure building residues in the membrane environment. On the one hand this suggests that SN3 in combination with PY3 is involved in global characterization of all investigated membrane protein families of DS2. On the other hand functional or structural characteristics accurately describe a family closer.To evaluate this assumption our approach has been applied to each single protein family of DS2. Out of the DS2 graph, information of two transmembrane receptor families (Pfam-Ids: PF00001, PF00002) has been compared with the results of an entropy based Profile Hidden Markov Model (pHMM)-alignment approach by [20] and colleagues. They present a visualization method that incorporates both emission and transition probabilities of the pHMM, thus extending sequence logos. Each protein family specific graph shows exactly the highly occurring motif combinations within the pHMM-alignments logos (see Figures 8 and 9). Further, networking with existing biological databases like PROSITE [21-24] delivers important information about protein domains, families and functional sites as well as associated patterns and profiles to identify them. In relation to PY3-SN3 (Pfam-Id: PF00001, Figure 10), supplied PROSITE information makes apparent, that these motifs are involved in consensus pattern of retinal binding sites [21-24] (PROSITE documentation PDOC00211) and thus are a significant figurehead for this Pfam receptor family. Analogously to this, NQ3-GI3 are also involved in consensus pattern of retinal binding sites [21-24] (PROSITE documentation PDOC00559) in Pfam family with Pfam-Id: PF00002 (Figure 11).

In summary, we could show that membrane protein families are characterized by individual motifs influenced by their structural and functional properties. Finally, on consideration of all data processing steps including by final visualising and under networking with biological databases, we are able to build a bridge between graph information in conjunction with a biological context.

## Conclusion

Generally, in this work it could have been shown how to visualize high-dimensional membrane protein data in form of graph structures and how to fill the lack between high-throughput protein data analyses and evaluation. 32 poly-topic membrane protein families with domains of unknown functions and 11 membrane protein families consisting of receptor, transporter and neurotransmitter-gated ion-channel proteins were analysed. Transmembrane and non-transmembrane sequence regions were predicted using the TMHMM method. Possible sequence motifs of variable lengths have been extracted out of predicted ‘TM’ regions, by using a naive text extracting algorithm. Four immediately consecutive sequence motifs were defined as a statistical frame called “motif-architecture”. Subsequently, multiple numbers of motif-architectures have been extracted out of all ‘TM’ regions, followed by information transformation into graph structures. Motifs as representative nodes connected by weighted edges to other nodes form a graph. All result graphs support the understanding and evaluation of high occurring consecutive motifs of the investigated protein families. This high occurrence of architecture-motifs points to the general importance that these motifs within the respective protein structure are significantly relevant for the membrane protein folding. ‘TM’ region atypical motifs have emerged which point to the general importance as being involved in defining a protein’s function. Here in special, motifs which are involved in the consensus pattern of retinal binding sites of Pfam receptor families. Finally, hub-motifs which often occur together with others point out to indispensable motifs in helical regions.

Because of the stronger protein structure conservation in evolution than the sequential composition of the folded protein chains, there are individual motifs or characteristic sequence parts which expose a certain biochemical function of proteins. This means that membrane protein families are characterized by structural and functional motifs. Thus, it is possible to compare such families by the inclusion of individual sequence motifs.

Conclusive evaluation of our results with biological databases confirms this fact and shows a simple way bridging visualisation of membrane protein data to biological context.

## Competing interests

The authors declare that they have no competing interests.

## Authors’ contributions

SG performed research and drafted the manuscript. Both authors read and approved the final manuscript.
